# To the Editors of the Medical and Physical Journal

**Published:** 1799-06

**Authors:** 


### *To the Editors of the Medical and Physical Journal.*

GENTLEMEN,

NOTWITHSTANDING the great improvements which have been made in the art of medicine, within the present century, there is one branch of it which has, till of late, been but little attended to—I refer to the treatment of incurable and mortal diseases: this is doubtless a subject of considerable importance: I shall therefore, without further apology, make a few observations on the treatment of a disease, which comes most unequivocally under the above description: I mean *aneurism of the aorta*, or of its more immediate branches. In cases of this kind, the practitioner has in general thought himself fully justified in leaving the patient quietly to his fate; but I was once witness to a case of aneurism of the right subclavian artery, in which the *digitalis* was from time to time exhibited in small doses, and I think the patient's life was obviously protracted by this mode of treatment. The well-known property which this drug possesses of lessening the force of the circulation, it may readily be supposed was the circumstance which induced the practitioner to make use of it on this occasion. Whether or not the practice is singular, I am unable to determine; but I think it is certainly worthy of imitation.

It unfortunately happens, that the case of aneurism of the aorta, or its more immediate branches, cannot always be ascertained during an early stage of the disease; where it can, however, I think the exhibition of the *digitalis*, together with an attention to avoid all causes which might hurry the circulation, would go near to check the progress of the tumor altogether, or at least render its growth very slow, and lengthen the life of the patient.

Should you think these hints worthy of being made public, they are very much at your service.

*London, May* 10, 1799. R. B. M.

---

